# Functional relevance of a six mesenchymal gene signature in epithelial-mesenchymal transition (EMT) reversal by the triple angiokinase inhibitor, nintedanib (BIBF1120)

**DOI:** 10.18632/oncotarget.4300

**Published:** 2015-05-27

**Authors:** Ruby Yun-Ju Huang, Kuee Theng Kuay, Tuan Zea Tan, Mohammad Asad, Hei Mui Tang, Aloysius Hsien Chun Ng, Jieru Ye, Vin Yee Chung, Jean Paul Thiery

**Affiliations:** ^1^ Department of Obstetrics and Gynaecology, National University Health System, Singapore; ^2^ Cancer Science Institute of Singapore, National University of Singapore, Singapore; ^3^ Dean's Office, Yong Loo Lin School of Medicine, National University of Singapore, Singapore; ^4^ Institute of Molecular and Cell Biology, A*STAR, Singapore; ^5^ Department of Biochemistry, National University of Singapore, Singapore

**Keywords:** epithelial-mesenchymal transition (EMT), siRNA screen, EMT reversal, nintedanib, triple angiokinase inhibitor

## Abstract

Epithelial-mesenchymal transition (EMT), a crucial mechanism in carcinoma progression, describes the process whereby epithelial cells lose their apico-basal polarity and junctional complexes and acquire a mesenchymal-like morphology. Several markers are considered to be authentic indicators of an epithelial or mesenchymal status; however, there is currently no comprehensive or systematic method with which to determine their functional relevance. Previously, we identified a 33-gene EMT signature comprising 25 epithelial and 6 mesenchymal genes that best describe this concept of the EMT spectrum. Here, we designed small-scale siRNA screens targeting these six mesenchymal signature genes (*CD99L2*, *EMP3*, *ITGA5*, *SYDE1*, *VIM*, *ZEB1*) to explore their functional relevance and their roles during EMT reversal by nintedanib (BIBF1120) in a mesenchymal-like SKOV3 ovarian cancer cell line. We found that neither cell proliferation nor cytotoxicity was affected by silencing any of these genes. SKOV3 cells expressing siRNA against mesenchymal genes (*ZEB1*, *EMP3*, *CD99L2*, *ITGA5*, and *SYDE1*) showed enhanced colony compaction (reduced inter-nuclear distance). Inductions of E-cadherin expression were only observed in *SYDE1*- and *ZEB1*-silenced SKOV3 cells. In addition, only *SYDE1*-silenced SKOV3 cells showed increased anoikis. Finally, we identified that *SYDE1* and *ZEB1* were down-regulated in nintedanib-treated SKOV3 cells and *SYDE1*- and *ZEB1*-silenced SKOV3 cells showed enhanced nintedanib-induced up-regulation of E-cadherin. Nintedanib-treated SKOV3 cells also showed colony compaction and decreases in EMT scores both *in vitro* and *in vivo*. We conclude that *SYDE1* and *ZEB1* are functionally relevant in EMT reversal. This study thus provides a proof-of-concept for the use of *in vitro* siRNA screening to explore the EMT-related functions of selected genes and their potential relevance in the discovery of EMT reversing drugs.

## INTRODUCTION

Epithelial-mesenchymal transition (EMT) describes the transdifferentiative process of epithelial cells into mesenchymal cells [1]. In terms of understanding cancer progression, the EMT process explains how *in situ* carcinoma cells transform the cellular state from epithelial to mesenchymal, with the cells transitioning through intermediate/metastable phases as they invade the local environment to metastasize [2, 3]. Transitioned carcinoma cells also acquire stem cell-like properties, which are reflected in their capacity to replicate seemingly uninhibited in the new location [2, 4, 5]. During carcinoma progression, cancerous cells are exposed to numerous EMT-inducing cues that lead to this acquisition of this transitioned or ‘EMTed’ phenotype [6]. The gain and/or loss of various molecules is regarded as being indicative of an EMTed phenotype [1, 7], with many of these molecules identified to be direct transcriptional targets of the EMT inducers SNAI and ZEB transcription factor family members [1]. With the exception of E-cadherin function—one of the best studied mechanisms in EMT—most of these EMT genes have not been extensively characterized for their functional relevance in terms of drug discovery or the regulatory pathways involved with their activity. During the course of EMT, E-cadherin is involved in the dynamic modulation of cell adhesion, which endows cells with altered migratory and invasive properties [8, 9].

Numerous EMT markers have been described, with almost 21 cancer-specific EMT signatures reported [10]. These EMT signatures show various degrees of correlation among each other, and, together, paint a picture of the continuous spectrum of EMT [10]. However, there has been limited comprehensive and systematic analysis to determine the functional relevance of each of the EMT markers derived from these EMT signatures. Previously, we defined EMT as a spectrum comprising four phenotypic subgroups—Epithelial (E), Intermediate E, Intermediate M, and Mesenchymal (M)—and uncovered a 33-gene EMT signature that best describes this phenotypic spectrum [6]. This gene list includes genes that are implicated in EMT as well as novel genes that have yet to be reported as having functional relevance in EMT. We have used this 33-gene EMT signature to classify an ovarian carcinoma (OC) cohort into different EMT states and correlated this classification with progression-free survival [6]. Our previous studies therefore suggest that these EMT genes can reflect certain functions related to the aggressiveness of carcinoma cells. It is uncertain though whether these EMT signature genes could serve as a potential readout or bear functional relevance in terms of reversing the EMT process.

EMT is reversible [3, 7], and this reversibility has emerged as an increasingly attractive, alternative therapeutic strategy for carcinoma [11] as compared with conventional cytotoxic agents that are aimed at eradication. Reversing EMT shares a similar concept to that of differentiation therapy [12]. However, robust models for the discovery of potential EMT reversal agents and their associated mechanisms are limited. An EMT model, established by silencing E-cadherin in human mammary epithelial cells (HMLE_shEcad), has been useful in screening for chemical compounds or target pathways that would have preferential cytotoxicity towards the cancer stem cell population in breast cancers [13, 14]. These studies, however, do not provide a working model that directly searches for non-cytotoxic EMT reversing agents. We have previously shown that a model incorporating the NBT-2 rat bladder carcinoma cell line offers a robust screening platform for the identification of EMT reversing agents [15]. The readout for this NBT-2 model is the inhibition of growth factor-induced cell scattering, which is an important phenotype during EMT. However, these models still cannot provide the mechanistic view to explain how EMT reversal was achieved.

To this end, here we outline a proof-of-concept for the use of six mesenchymal genes derived from the 33-gene EMT signature in their functional relevance to EMT reversal. We designed small-scale siRNA screens to explore the *in vitro* functions of these six mesenchymal EMT genes using four assays—cytotoxicity, colony compaction, induction of E-cadherin gene expression, and anoikis resistance. Furthermore, we explored the functional contribution of these six mesenchymal genes in the presence of an EMT reversing agent, nintedanib (BIBF1120). Our findings demonstrate the utility of this six-mesenchymal gene signature in ascertaining relevant functions during EMT reversal.

## RESULTS

### The six-gene mesenchymal signature is correlated with disease outcomes

**Figure 1:**
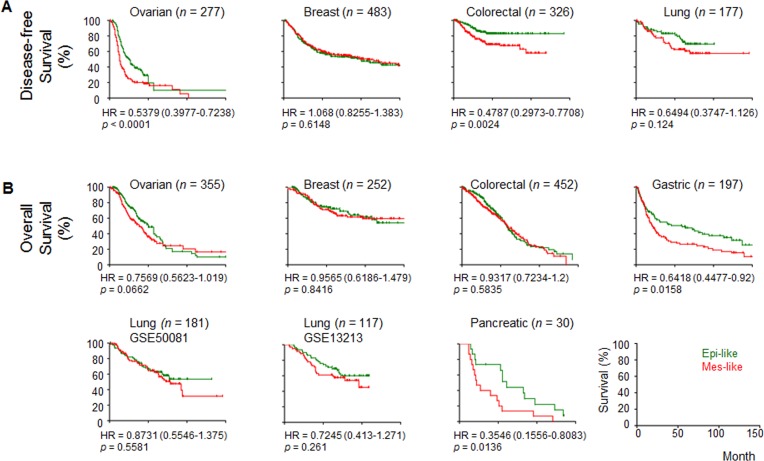
Correlation of the epithelial-mesenchymal transition (EMT) status with survival in cancers Kaplan-Meier analyses of the EMT status, as estimated by average expression of CD99L2, EMP3, ITGA5, SYDE1, VIM, and ZEB1 with **A.** disease-free survival and **B.** overall survival. Disease-free survival includes progression- and recurrence-free survival. Samples were stratified into (Epi)thelial- (green) or (Mes)enchymal-like (red) groups based on the median average expression from the six genes. Hazard ratio (HR) ± 95% confidence interval and *p*-value from log-rank test are given.

We validated the expression levels of the 33 genes from the EMT signature [6] on a panel of OC cell lines, SGOCL(43), and confirmed that six mesenchymal genes showed good correlation with mesenchymal(Mes)-like phenotypes (for details, refer to Supplementary Materials). We first explored whether this six-mesenchymal gene signature showed any clinical relevance using microarray datasets across various cancer types; this analysis was chosen to assign Epi-like or Mes-like subgroups and correlate the subgroups with survival (Figure 1). For progression/disease-free survival (PDFS), which includes progression-free and recurrence-free survival rates, the Mes-like subgroups showed significantly shorted PDFS in the ovarian and colorectal cancer datasets (Figure 1A). For overall survival (OS), the Mes-like subgroups showed significantly poorer outcomes in the ovarian, gastric, and pancreatic cancers (Figure 1B). This result was concordant with our previous report on the correlation between EMT status defined by a generic EMT signature and survival outcomes [10]. Our analysis indicates the strong clinical importance of the six-mesenchymal gene signature and therefore warrants further understanding of their functional relevance.

### Mesenchymal signature genes have little effect on proliferation

**Figure 2:**
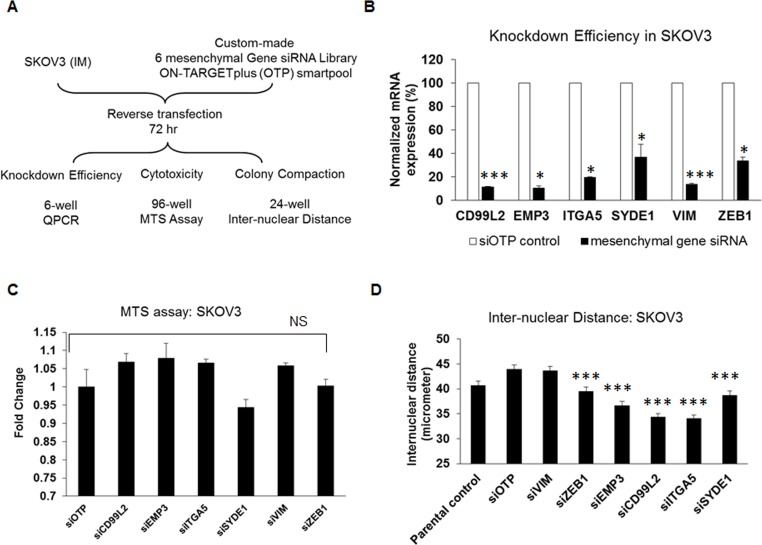
Effect of siRNAs against the six mesenchymal signature genes on cytotoxicity and colony compaction **A.** A flowchart of the experimental design of the siRNA screens for the six mesenchymal signature genes in Intermediate Mesenchymal (IM) SKOV3 cells. **B.** Plot of the fold change in expression (*y*-axis) of target genes (*x*-axis) following the transient knockdown of mesenchymal genes (black bars) as compared with the siOTP control (white bars) in SKOV3 cells. **C.** Plot of the fold change of MTS readouts (*y*-axis) following the transient knockdown of mesenchymal genes and siOTP control (*x*-axis) in SKOV3 cells. **D.** Plot of the inter-nuclear distance (*y*-axis) following the transient knockdown of mesenchymal genes in SKOV3 cells. The *x*-axis represents SKOV3 parental, siOTP control, and six mesenchymal genes. Error bars indicate the S.E.M from 200 random nuclei. *p < 0.05; ***p < 0.005; NS = not significant.

We were interested to know whether these six mesenchymal signature genes would have functional relevance in reversing EMT. Assays were designed using a custom-made siRNA library for the six-mesenchymal signature genes, assembled into 6-well, 96-well and 24-well formats, to test the knockdown efficiency and the effects of these genes on cell proliferation (cytotoxicity) and morphology (colony compaction) (Figure 2A). The optimization of this siRNA screen is described elsewhere [16]. The knockdown efficiency of these six mesenchymal genes ranged from 63% to 88% (Figure 2B). We first examined the effects of these six mesenchymal genes on cell proliferation using an MTS assay 72 hours post-transfection in the Intermediate Mesenchymal SKOV3 cell line. As shown in Figure 2C, the transient knockdown of the mesenchymal genes in SKOV3 cells showed no significant effect on cell proliferation as compared with the negative control siRNA (Figure 2C, Table 1). Thus, our data suggest that these mesenchymal signature genes had minimal to no effect on cell proliferation or cytotoxicity.

**Table 1:** Summary of functional relevance of six mesenchymal genes

		Transient Silencing in SKOV3				OC Molecular Subtype *p*-value^^^^				
Symbol	Entrez Gene Name	Proliferation Ratio^^^	Inter-nuclear Distance (∆ Ratio^^^)	CDH1 QPCR Ratio^^^	E-cad Protein Ratio^^^	Epi-A vs Rest	Epi-B vs Rest	Mes vs Rest	Stem-A vs Rest	Stem-B vs Rest
*CD99L2*	CD99 molecule-like 2	1.069	−0.217***	1.299	1.21	3.84e-01	1.08e-02	2.93e-03	3.63e-01	2.58e-01
*EMP3*	epithelial membrane protein 3	1.079	−0.166***	1.114	1.39	7.07e-10	9.85e-01	5.20e-56	4.48e-24	5.41e-04
*ITGA5*	integrin, alpha 5	1.066	−0.224***	0.956	1.43	1.25e-08	9.70e-15	1.27e-77	2.11e-21	6.11e-02
*SYDE1*	synapse defective 1, Rho GTPase, homolog 1 (C. elegans)	0.943	−0.118***	1.584*	2.72*	3.38e-01	6.90e-10	1.14e-18	1.69e-03	2.81e-01
*VIM*	vimentin	1.058	−0.007	0.803	1.42	5.72e-02	4.72e-35	9.35e-18	3.79e-01	9.41e-06
*ZEB1*	zinc finger E-box binding homeobox 1	1.003	−0.100***	4.985***	23.00***	3.32e-05	1.20e-29	3.05e-56	9.95e-10	7.12e-07

Abbreviations: OC, ovarian cancer; Epi-A, epithelial type A; Epi-B, epithelial type B; Mes, mesenchymal; Stem-A, Stem cell-like A; Stem-B, stem cell-like B.

^ Ratio between siOTP control and si-mesenchymal genes.

^^ *p*-value by Mann–Whitney Test

* *p* < 0.05;

**p < 0.01;

*** *p* < 0.005 (*t*-test).

### Colony compaction is increased by the inhibition of mesenchymal signature genes

Colony compaction is one of the hallmarks of epithelial-like cells. We next investigated the effect of knocking down the six mesenchymal signature genes on colony compaction by examining the inter-nuclear distances. As shown in Figure 2D, the transient knockdown of five out of the six mesenchymal genes in SKOV3 cells resulted in significantly decreased inter-nuclear distances. Whereas the negative control cells showed an average inter-nuclear distance of 43.9 μm, the si*ZEB1*-, si*EMP3*-, si*CD99L2*-, si*ITGA5*-, and si*SYDE1*-SKOV3 cells showed significantly reduced mean inter-nuclear distances of 39.5, 36.6, 34.4, 34.0, and 38.7 μm, respectively (*p* = 0.0002; 4.02E-09; 8.52E-17; 2.28E-18; 3.76E-05). Cells treated with si*VIM* had an average inter-nuclear distance of 43.6 μm, demonstrating that transient knockdown of *VIM* had no effect on colony compaction in SKOV3 cells. The degree of colony compaction in response to these siRNAs ranged from 10% to 22% (Table 1). Overall, our data suggest that, by manipulating the expression of certain mesenchymal genes, the EMT phenotype can be partially altered.

### Restoring E-cadherin expression by inhibiting *SYDE1* and *ZEB1* mesenchymal signature genes

**Figure 3:**
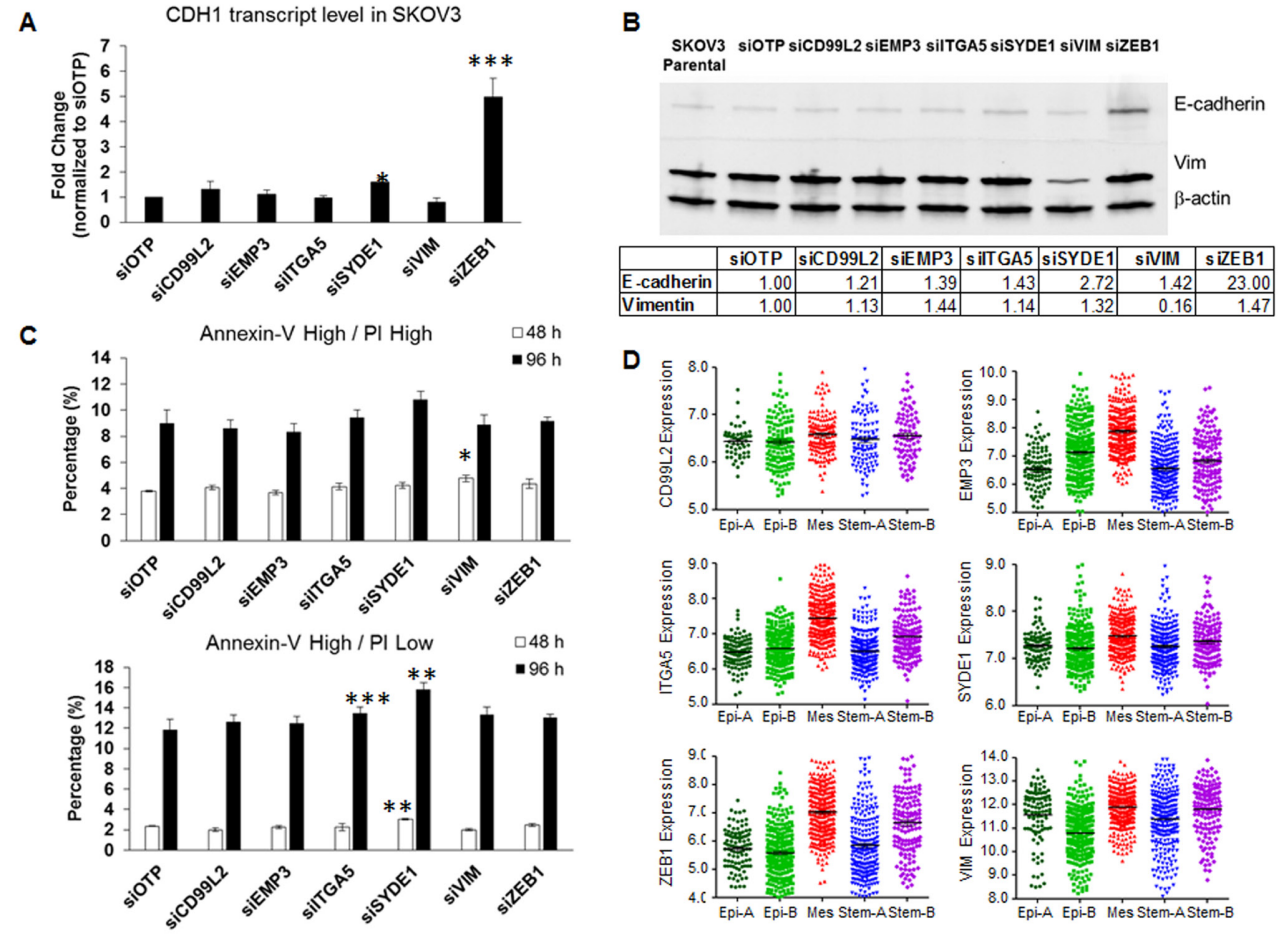
Effects of siRNA against the six mesenchymal signature genes on CDH1 expression, E-cadherin protein expression, and anoikis resistance **A.** Plot of the fold change of *CDH1* expression (*y*-axis) following the transient knockdown of mesenchymal genes and OTP control (*x*-axis) in SKOV3 cells. **B.** Western blots of E-cadherin (upper panel), vimentin (Vim, middle panel), and β-actin (lower panel) in SKOV3 parental, OTP negative control, and cells harboring a transient knockdown of each of the six mesenchymal genes. Quantitative readings from densitometry are shown in the table below. **C.** Plots of the percentage (*y*-axis) of cells in the Annexin-V^high^/PI^high^ (upper) and Annexin-V^high^/PI^low^ (lower) populations following the transient knockdown of the six mesenchymal genes and OTP control (*x*-axis) in SKOV3 cells. **D.** Gene expression data of the six mesenchymal signature genes from 1,538 ovarian tumor samples grouped into five, biologically distinct subgroups: Epithelial (Epi)-A, Epi-B, Mesenchymal (Mes), Stem cell-like type A (Stem-A) and Stem-B. *p < 0.05; **p < 0.01; ***p < 0.005.

Despite observing significant colony compaction upon knocking down mesenchymal signature genes, we did not observe any full reversal of EMT phenotypes under the conditions tested (Supplementary Figure 2). This prompted us to examine if this partial EMT reversal was associated with the restoration of the prototypic epithelial gene, E-cadherin/*CDH1*. Only si*SYDE1*- and si*ZEB1*-SKOV3 cells showed a significant up-regulation in *CDH1*, up to 1.58- (*p* = 0.026) and 4.99-fold (*p* = 0.0008), respectively (Figure 3A & Table 1). E-cadherin protein expression was confirmed by western blotting, which showed a similar trend, with increased E-cadherin protein expression found in si*SYDE1*- and si*ZEB1*-SKOV3 cells (Figure 3B). Our data therefore indicate that *SYDE1* and *ZEB1* act upstream E-cadherin and its expression.

### Anoikis resistance is altered by the inhibition of mesenchymal signature genes

The reversal of EMT has been shown to correlate with altered anoikis resistance [6]. We thus also sought to explore whether these six mesenchymal signature genes would contribute to anoikis resistance. We compared the percentage of the Annexin-V-positive populations at 48 h and 96 h in ultra-low attachment cultures, with this time difference use to assess any increments in cells entering apoptosis. For the cell fractions entering the early anoikis phase (Annexin-V^high^/PI^high^ population), we found no significant difference in anoikis resistance, with the exception of a slight increase for the si*VIM* cells at 48 h as compared with the siOTP control (Figure 3C, upper). For cells in the late anoikis phase (Annexin-V^high^/PI^low^ population), we measured a significant increase in the si*SYDE1* cells at both 48 h (2.35 % to 3.05 %) and 96 h (11.85 % to 15.85 %) (Figure 3C, lower), as compared to the siOTP control. The si*ZEB1* cells did not show significant increase in Annexin-V^high^ populations. These results suggest that, between the two genes that regulate E-cadherin expression, *SYDE1* further regulated anoikis resistance ability.

### Mesenchymal signature genes are overexpressed in the Mes molecular subtype of OC

Since the six-mesenchymal gene signature predicted worse PDFS and OS in OC (Figure 1), we validated the expression patterns of these six mesenchymal genes in a gene expression profiling meta-analysis of 1,538 OC samples [16]. As shown in Figure 3D, these six mesenchymal genes were all overexpressed in the Mes molecular subtype of OC, a subtype designated as having the worst OS. A binary comparison between the Mes subtype versus the other subtypes using the Mann-Whitney Test showed the most extreme significance of the overexpression of *EMP3*, *ITGA5*, *SYDE1*, and *ZEB1* in the Mes molecular subtype (Table 1). Since these six mesenchymal genes had minimal roles on cell proliferation, we concluded that the aggressiveness of the Mes molecular subtype might not be due to preferential growth advantage. Instead, this aggressiveness might be attributed to the ability of the EMT phenotype to respond to changes in the microenvironment, such as overcoming anoikis.

### Triple angiokinase inhibitor, nintedanib, restores E-cadherin expression during EMT reversal

**Figure 4:**
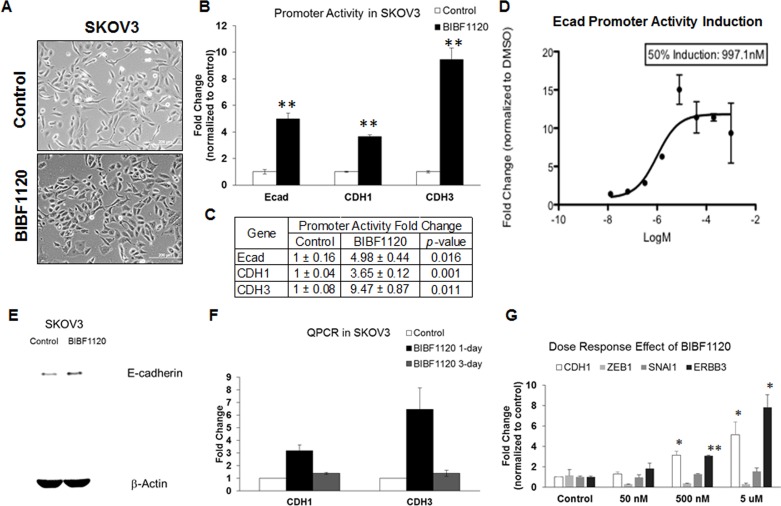
Effect of nintedanib treatment on the expression levels and promoter activities of E-cadherin in SKOV3 cells **A.** Phase contrast images of SKOV3 in DMSO (upper panel) or 5 μM nintedanib (BIBF1120) (lower panel)-treated cultures. Bar: 100 μm. **B.** Plot of E-cadherin (Ecad), CDH1, and CDH3 promoter activity fold change (*y*-axis) in control (open bars) or 5 μM nintedanib (BIBF1120) (black)-treated SKOV3 cells. **p < 0.01. **C.** Summary of quantitative E-cadherin (Ecad), CDH1, and CDH3 promoter activity fold change. **D.** Dose-dependent curve of E-cadherin (Ecad) promoter activity induction fold change (*y*-axis) of various concentrations of nintedanib (*x*-axis). **E.** Western blots of E-cadherin (upper panel) and β-actin (lower panel) in control or 5 μM nintedanib (BIBF1120)-treated SKOV3 cells from a whole gel image. **F.** Plot of the fold change (*y*-axis) for CDH1 and CDH3 in the control or nintedanib (BIBF1120)-treated SKOV3 cells for 1 day (black bars) and 3 days (grey bars). **G.** Plot of dose-dependent fold change (*y*-axis) of CDH1 (white bars), ZEB1 (light grey bars), SNAI1 (dark grey bars) and ERBB3 (black bars) in the control or 50 nM, 500 nM, 5 μM nintedanib (BIBF1120) (*x*-axis). Error bars indicate SEM from three independent experiments. *p < 0.05.

EMT reversal can be achieved by tyrosine kinase inhibitors (TKIs), such as the Src-kinase inhibitor saracatinib (AZD0530), which acts to up-regulate E-cadherin expression both *in vitro* and *in vivo* [6]. Here, we treated Intermediate Mesenchymal SKOV3 cells with nintedanib (BIBF1120), another TKI that has phenotypic EMT reversing effects (Figure 4A). We have previously demonstrated that a short version of the E-cadherin promoter region containing the E-box sequences can be used to reflect an increase in *CDH1* promoter activity upon EMT reversal [6]. *CDH1* maps to the human chromosome 16q22.1 and is in tandem with *CDH3*, the gene coding for P-cadherin, which shares similar control at the upstream promoter region [24]. To test if nintedanib would have the same induction of *CDH1* promoter activity, we cloned the putative 1.2-kb promoter region around the transcriptional start site (TSS) (Supplementary Table 1) for *CDH1* and *CDH3* into a pGL3basic luciferase expression vector. Transient transfection was performed with the control (pGL3basic) or the shorter (E-cad) or full 1.2-kb *CDH1* and *CDH3* promoter constructs, and SKOV3 cells were then treated with DMSO or 5 μM nintedanib for 24 h. Using the dual-luciferase assay, we found that nintedanib induced a significant increase in the promoter activities of E-cad, *CDH1*, and *CDH3* (Figure 4B, 4C), and that this increase linearly correlated with nintedanib concentrations; indeed, a 50% induction of E-cad promoter activity was determined at about 1 μM, with induction plateauing at 5 μM (Figure 4D). Furthermore, 5 μM nintedanib concomitantly up-regulated both E-cadherin protein and *CDH1* transcript levels (Figure 4E, 4F). In a wash-out experiment after 5 μM nintedanib for 24 h or prolonged treatments for up to 3 days without replenishment, we noted that the mean fold-change in *CDH1* transcript levels dropped between 1.4–1.5-fold (Figure 4F). Finally, consistent with the promoter activity results, the induction of *CDH1* gene expression followed a dose-dependent trend (Figure 4G). Collectively, these data indicate that E-cadherin gene induction follows a dose-dependent, linear function of the active concentration of nintedanib, and we can therefore conclude that EMT reversal and E-cadherin restoration caused by nintedanib were not due to random off-target effects.

### *ZEB1* mediates the nintedanib-induced E-cadherin restoration and EMT reversal

**Figure 5:**
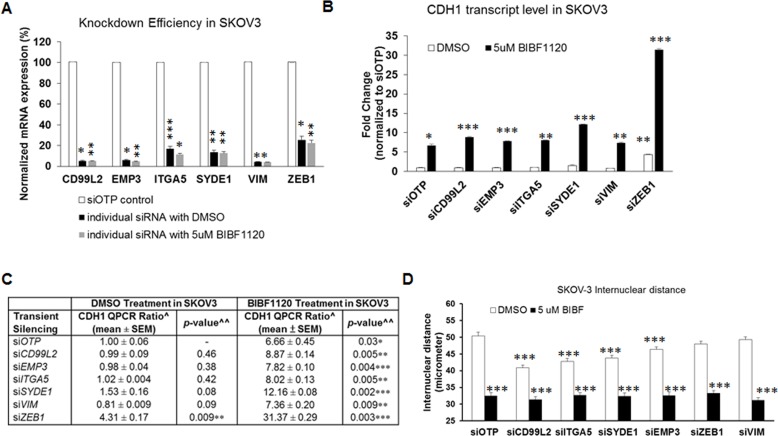
Effects of six mesenchymal signature genes on the nintedanib-induced EMT reversal **A.** Plot of the fold change of the expression (*y*-axis) of target genes (*x*-axis) following the transient knockdown of mesenchymal genes treated with DMSO (grey bars) or 5 μM nintedanib (BIBF) (black bars) compared to the siOTP control (white bars) in SKOV3 cells. **B.** Plot of the fold change of CDH1 expression (*y*-axis) following the transient knockdown of mesenchymal genes and OTP control treated with DMSO (white bars) or 5 μM nintedanib (BIBF) (*x*-axis) in SKOV3 cells. **C.** Summary of quantitative CDH1 fold change gene expression. ^Ratio between DMSO-treated or BIBF1120-treated siOTP control and si-mesenchymal genes. ^^T-test statistics compared to DMSO-treated siOTP control. **D.** Plot of the inter-nuclear distance (*y*-axis) following the transient knockdown of mesenchymal genes in SKOV3 cells. *p < 0.05; **p < 0.01; ***p < 0.005.

We continued to explore the possible mechanism of nintedanib-mediated E-cadherin induction by examining changes in the expression of several known EMT players: *CDH1*, *ERBB3*, *SNAI1*, and *ZEB1*. The induction of epithelial genes *CDH1* and *ERBB3* followed a dose-dependent trend following nintedanib treatment (Figure 4G). However, nintedanib only caused a significant reduction in *ZEB1* but not *SNAI1* expression (Figure 4G); this is different from saracatinib, which reduces *SNAI1* but not *ZEB1* expression [6]. Interestingly, nintedanib also induced a 2-fold down-regulation in the mesenchymal gene *SYDE1* (data not shown). We next asked whether the EMT reversing effect of nintedanib might be mediated by mesenchymal signature genes other than *ZEB1*. We utilized a custom-made siRNA library for the six mesenchymal signature genes to test the differences in the nintedanib effects on E-cadherin induction (Figure 5A). Consistent with the siRNA-only results, si*SYDE1* and si*ZEB1* increased *CDH1* expression by 1.5- and 4.3-fold, respectively, in DMSO-treated SKOV3 cells (Figure 5B, 5C). As expected, nintedanib treatment caused significant *CDH1* induction in all siRNA-treated groups as compared with the DMSO control. The nintedanib-treated si*SYDE1* and si*ZEB1* SKOV3 cells showed the highest *CDH1* induction at 12.1- and 31.4-fold, respectively (Figure 5B, 5C). These data indicate that nintedanib alone might act through the down-regulation of the mesenchymal genes, *SYDE1* and *ZEB1*, to directly restore *CDH1* expression. Further silencing of *SYDE1* and *ZEB1* might sensitize the cells for nintedanib-induced EMT reversal.

Interestingly, nintedanib-treated si*CD99L2*, si*EMP3*, siITGA5, and si*VIM* SKOV3 cells also showed slightly higher *CDH1* induction (8.87-, 7.82-, 8.02-, 7.36-fold, respectively) compared with the siOTP control (6.66-fold) (Figure 5C). This suggests that transient silencing of these mesenchymal signature genes further sensitizes the nintedanib-induced *CDH1* induction in these Intermediate Mesenchymal SKOV3 cells. The expression of *SYDE1* and *ZEB1* remained unchanged (data not shown) under these conditions; therefore, we speculate that the mechanisms might be *SYDE1*- and *ZEB1*-independent for these siRNAs. In terms of colony compaction, as measured by inter-nuclear distance, nintedanib treatment did not induce further changes following silencing of all six mesenchymal genes as compared with the siOTP control (Figure 5D), suggesting that nintedanib alone causes colony compaction effects independent of the induction of *CDH1* expression.

### Combination of EMT reversing TKIs significantly enhances E-cadherin restoration but causes cell-cycle arrest

**Figure 6:**
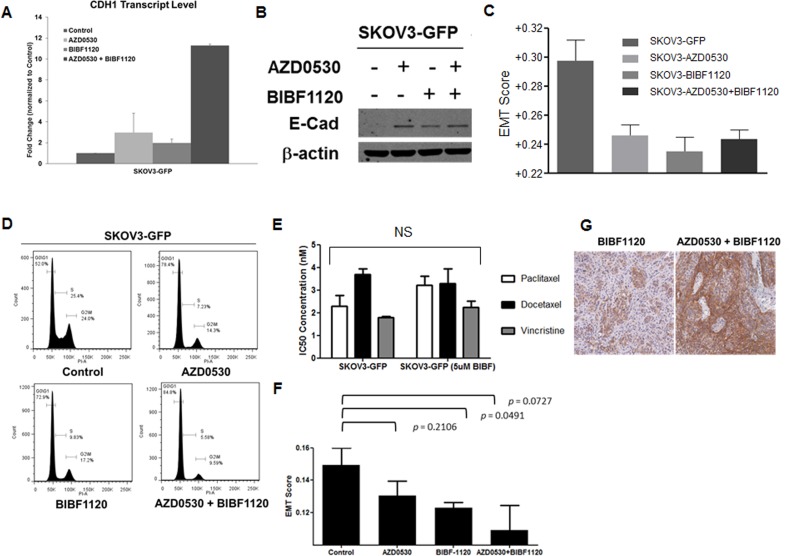
Effects of combination of saracatinib and nintedanib in EMT reversal **A.** Plot of the fold change (*y*-axis) for *CDH1* in the control or saracatinib (AZD0530)-, nintedanib (BIBF1120)-, or combined (AZD0530+BIBF1120)-treated SKOV3-GFP cells. **B.** Western blots of E-cadherin (E-Cad, upper panel) and β-actin (lower panel) in control or saracatinib (AZD0530)-, nintedanib (BIBF1120)-, or combined (AZD0530+BIBF1120)-treated SKOV3-GFP cells. **C.** Plot of the EMT scores (*y*-axis) of SKOV3-GFP cells treated with control (SKOV3_GFP), saracatinib (SKOV3_AZD), nintedanib (SKOV3_BIBF), or combined (SKOV3_AZD+BIBF). **D.** Cell cycle analysis of SKOV3-GFP cells treated with control, saracatinib (AZD0530), nintedanib (BIBF1120), or combined (AZD0530+BIBF1120). **E.** Plot of the 50% growth inhibitory concentration (IC50, *y*-axis) in SKOV3-GFP cells treated with paclitaxel (white bars), docetaxel (black bars), and vincristine (grey bars) with or without nintedanib (5 μM BIBF). **F.** Plot of the EMT scores of SKOV3-luc-D3 xenografts treated with control, saracatinib (AZD0530), nintedanib (BIBF1120), or combined (AZD0530+BIBF1120). **G.** Representative images of immunohistochemistry staining of E-cadherin in BIBF1120 or AZD0530+BIBF1120 treated SKOV3-luc-D3 xenografts.

We went on to test if the combination of EMT reversing TKIs would have a synergistic effect on EMT reversal and E-cadherin restoration. SKOV3 cells were labeled with green fluorescence protein (GFP) and then treated with saracatinib and nintedanib as single agents or in combination (double TKI or dTKI). As shown in Figure 6, the combination of 2.5 μM saracatinib and 2.5 μM nintedanib significantly increased *CDH1* transcript levels as compared with 5 μM of each agent alone (Figure 6A). However, E-cadherin protein levels did not show further enhancement (Figure 6B). To confirm whether the combination could further change the EMT status of SKOV3-GFP cells, we used gene expression microarray analysis, and derived quantitative EMT scores for these cells as described previously [10]. The dTKI-treated SKOV3-GFP cells demonstrated a trend toward a decrease in EMT scores as compared with the control (Figure 6C). Nintedanib-treated SKOV3-GFP cells showed a slightly lower EMT score as compared with those treated with saracatinib or both drugs in combination. The effect of saracatinib-nintedanib combination on E-cadherin promoter activity was further assessed based on Chou-Talalay combination index (CI) [30]. The CI analysis showed that saracatinib-nintedanib combination indeed had a synergistic effect (Supplementary Figure 3B, 3C). We also noticed that these dTKI-treated cells showed slower propagation rates *in vitro* and, upon performing cell cycle analysis, these dTKI-treated cells demonstrated significantly decreased G2/M and increased G0/G1 populations (Figure 6D). The gene ontology (GO) analysis from the expression microarray data of these dTKI-treated cells also supported our findings (Supplementary Figure 3A). Therefore, we suspected that these dTKI-treated cells might not have enhanced sensitivity towards conventional chemotherapeutics, such as mitotic inhibitors. As shown in Figure 6E, the GI50 of nintedanib-treated SKOV3-GFP cells to paclitaxel, docetaxel and vincristine did not differ much from the DMSO-treated cells. Therefore, we concluded that these TKIs with EMT reversing effects, as single agents or in combination, might not be ideal to induce chemo-responsiveness or to reverse chemo-resistance. However, we did observe that when treating the SKOV3 cells *in vivo* xenografts with saracatinib and nintedanib as single agents or in combination, the EMT scores of the xenografts were decreased, suggesting a reversal (Figure 6F). In addition, these dTKI-treated xenografts demonstrated enhanced resectability during tumor harvesting, suggesting further modifications to the tumor microenvironments.

### Nintedanib-induced EMT reversal in selected lung, bladder, and pancreatic cancer cells

**Figure 7:**
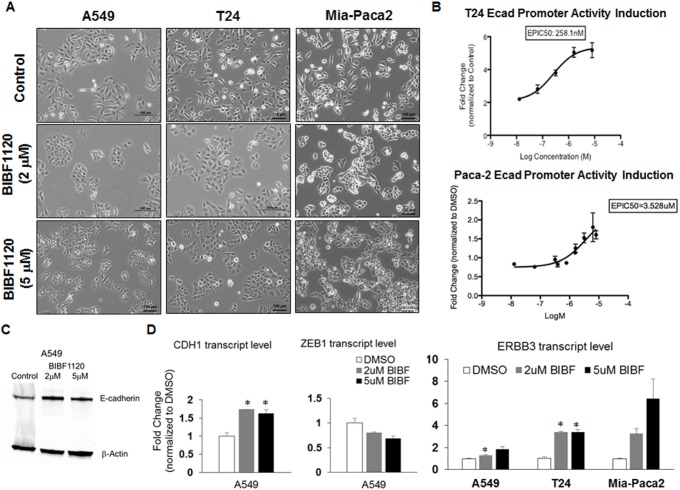
Effect of nintedanib treatment on the expression levels and promoter activities of E-cadherin in A459, T24, and Mia-Paca2 cells **A.** Phase contrast images of A549, T24, and Mia-Paca2 cells in DMSO (upper panel) or 2 (middle panel) and 5 μM nintedanib (BIBF1120) (lower panel)-treated cultures. Bar: 100 μm. **B.** Dose-dependent curve of E-cadherin (Ecad) promoter activity induction fold change (*y*-axis) of various concentrations of nintedanib (*x*-axis). **C.** Western blots of E-cadherin (upper panel) and β-actin (lower panel) in control or 2 and 5 μM nintedanib (BIBF1120)-treated A549 cells from a whole gel image. **D.** Plot of dose-dependent fold change (*y*-axis) of *CDH1*, *ZEB1*, and *ERBB3* in the control (white bars) or 2 μM (grey bars), 5 μM (black bars) nintedanib (BIBF) in A549, T24, and Mia-Paca2 cells (*x*-axis). Error bars indicate SEM from three independent experiments. *p < 0.05.

To test if the nintedanib effect in EMT reversal is exclusive for ovarian cancer, three additional cell lines A549, T24, and Mia-Paca2 representing lung, bladder, and pancreatic cancer were selected for testing. These three cell lines were selected due to their similar EMT scores to SKOV3 [10] suggesting that they are at a similar intermediate EMT state. As shown in Figure 7A, all three cell lines demonstrated significant colony compaction and reversal to a more epithelial phenotype. The bladder adenocarcinoma cell line T24 and the pancreatic adenocarcinoma cell line Mia-Paca2 showed induction of E-cadherin promoter activity upon nintedanib treatment (Figure 7B). The 50% required doses for E-cadherin promoter induction concentration (EpIC-50) identified for nintedanib to restore E-cadherin expression in both T24 and Mia-Paca2 cells were 260 nM and 3.5 μM, respectively (Figure 7B). In A549 cells, both the transcript and protein expressions of E-cadherin were significantly increased following nintedanib treatment (Figure 7C, 7D). The increase of *CDH1* transcript in A549 cells was negatively correlated with the decrease of *ZEB1* expression (Figure 7D). In concordance with SKOV3 cells, the expressions of another epithelial marker ERBB3 were also significantly induced in A549 and T24 cells and had an increasing trend in Mia-Paca2 cells (Figure 7D). Our data supported that nintedanib had a general EMT reversal effect on cancer cell lines with an intermediate EMT state in the *in vitro* system.

## DISCUSSION

Various EMT markers have been touted as reliable indicators of an epithelial or mesenchymal status; yet, their functional relevance along the EMT spectrum has not been clearly established. In this study, we provide a proof-of-concept for the use of *in vitro* siRNA screening to ascertain the role of these indicators in EMT-related functions. Using six mesenchymal genes from a previously established 33-gene EMT signature, we identified that transient silencing of mesenchymal genes *ZEB1*, *EMP3*, *CD99L2*, *ITGA5*, and *SYDE1* reduced the inter-nuclear distance in SKOV3 cells without affecting cell proliferation, thereby causing a partial EMT reversal. Furthermore, we show that reversing the EMT phenotype in SKOV3 cells with nintedanib was accompanied by an up-regulation of *CDH1*, at both the gene expression level and at the promoter region, as well as a down-regulation of the mesenchymal genes, *SYDE1* and *ZEB1*. Down-regulating these two mesenchymal genes, *SYDE1* and *ZEB1*, further enhanced the effect of nintedanib in *CDH1* induction, suggesting that nintedanib might act via *SYDE1* and *ZEB1* to mediate E-cadherin restoration and EMT reversal effects.

SKOV3 cells transiently silenced with siRNA against *CD99L2* and *ITGA5* showed the greatest reduction (∼20%) in inter-nuclear distance and, consequently, increase in colony compaction. *CD99L2* is a much less-studied gene as compared with *ITGA5* yet they are both known to mediate cell adhesion. *CD99L2* encodes a cell-surface protein that is similar to CD99 and plays a role in the later stages of leukocyte extravasation across the endothelial basement membrane [17]. *ITGA5* encodes the integrin alpha 5 chain, which undergoes post-translational cleavage in the extracellular domain to yield disulfide-linked light and heavy chains that join with beta 1 to form the fibronectin receptor [18]. α5β1 integrin has an unambiguous role in promoting angiogenesis and carcinoma metastasis in ovarian and lung carcinoma, glioblastoma, and melanoma [19]. Patients with high α5β1 integrin expression are often associated with poorer outcomes. From our dataset, the *ITGA5* expression level was also significantly higher in the Mes molecular subtype of OC, which is correlated with worse survival. Therefore, it has become an attractive therapeutic target in solid tumors, with various therapeutic approaches, including humanized antibodies, non-peptide antagonists with RGD-like motifs, and non-RGD like peptides, which interfere with integrin binding [19].

We identified *SYDE1* and *ZEB1* to be the downstream mediators for nintedanib-induced *CDH1* (E-cadherin) restoration. The transcriptional control of *CDH1* by *ZEB1* has been extensively demonstrated in various settings during EMT [2]. There has been no previous association that *SYDE1* might play a role in the transcriptional control of *CDH1*. *SYDE1*, the gene coding for Synapse Defective Protein 1 Homolog 1, is the human ortholog for syd-1 in *C. elegans* and *Drosophila*. Syd-1 is an important regulator in pre-synaptic active zone assembly [20, 21]. *SYDE1* was included in a prognosis-associated 3-gene signature in renal cell carcinoma [22]. Its functional roles in mammals are not clear. Transient silencing of *SYDE1* and *ZEB1* significantly enhanced the nintedanib-induced *CDH1* transcript expression. This suggests that down-regulation of *SYDE1* and *ZEB1* might “prime” the cells towards the epithelial side of the spectrum to become more “Intermediate Epithelial”-like, and this, in turn, sensitizes the cell's response to nintedanib in terms of EMT reversal. Intriguingly, nintedanib has been shown to exert no effect on either the EMT phenotype or E-cadherin expression in lung or pancreatic cancer xenograft models [23]. In our hands, SKOV3 xenografts have shown a significant induction of E-cadherin expression and decreased EMT scores following nintedanib treatment. The fact that nintedanib initiated a dose-dependent induction of E-cadherin promoter activity suggests that the effect is unlikely to be a random, off-target effect. We further demonstrated that this reversal can be applied to other *in vitro* cell lines in lung, bladder, and pancreatic cancers. Therefore, the transcriptional control of the E-cadherin gene at the proximity of the promoter site provides the crucial key to understanding reversibility.

In humans, *CDH1* is located on the plus strand of chromosome 16q22.1 and is arranged in tandem with the P-cadherin gene, *CDH3*, which is situated 14-Kb upstream of *CDH1*. We identified that *CDH1* and *CDH3* were both up-regulated following nintedanib treatment. We previously reported that E-cadherin expression can be restored in SKOV3 cells by Saracatinib (AZD0530) [6]. *CDH3* promoter activity was also significantly enhanced in SKOV3 cells following saracatinib treatment (unpublished data). The promoter regions of *CDH1* and *CDH3* are highly conserved, harboring a GC-rich region with an SP1 binding site, a CAAT box, and two AP2 binding motifs without a TATA box. It is worth noting that the E-box sequences are only located in the *CDH1*, not *CDH3*, promoter region [24]; thus, *CDH1* transcription can be negatively regulated by the binding of the SNAI and ZEB transcriptional repressor families to the E-boxes [25]. Data elucidating the transcriptional control of *CDH3* at its promoter, however, has been limited. The expression of *CDH1* and *CDH3* in the OC cell line panel, SGOCL(43), were highly correlated with an Epithelial phenotype, with the highest expression occurring at this stage of the EMT spectrum (Supplementary Figure 1). *CDH1* and *CDH3* showed a concordant up-regulation following nintedanib treatment in SKOV3 cells, which have a low basal expression of *CDH1* and *CDH3*. This suggests that there could be a synchronized effect on the common regions within the 1.2-kb promoter sequences. One possibility would be that the regulation of *CDH1* and *CDH3* promoter activation by nintedanib is independent of E-boxes because of its absence in the *CDH3* promoter region. Since we also observed a similar increase in the promoter activity using the short version of the E-cadherin promoter (−108 to +125), we speculate that the responsive elements of nintedanib must be within the overlapping 207-bp (−108 to +99) region of the E-cadherin gene. Another possibility is that the regulation of *CDH1* promoter activation still depends on E-boxes and the *CDH3* promoter activation follows a separate mechanism. This could be supported by the suppression of *ZEB1* following nintedanib treatment. Nevertheless, the functional consequence of the concordant up-regulation of *CDH1* and *CDH3* warrants further investigation. Many studies have elucidated the potential roles of P-cadherin in interfering with E-cadherin functions and in facilitating metastasis [26-28]. Therefore, relying on one marker (such as E-cadherin) alone to design a screening platform for EMT reversing agents might not be optimal. Based on the unique similarity of the promoter structure between *CDH1* and *CDH3*, we propose prospectively a high-throughput screening (HTS) model utilizing the *CDH1* and *CDH3* promoter activities as the major readouts to screen for EMT reversing compounds that would only preferentially induce *CDH1* expression.

The increased induction of E-cadherin gene expression following the co-treatment of nintedanib with siRNAs against the six mesenchymal genes reveals that transient silencing of these mesenchymal signature genes further sensitized the nintedanib-induced *CDH1* induction. It is clear that *SYDE1* and *ZEB1* might be the crucial regulatory nodes mediating the nintedanib effect in *CDH1* induction. Further depletion of either *SYDE1* or *ZEB1* significantly enhances the nintedanib-induced *CDH1* up-regulation, suggesting that the restoration of E-cadherin expression or reversal of EMT can be achieved more efficiently by targeting the pivotal regulatory nodes. The induction of *SYDE1* or *ZEB1* downstream of nintedanib might explain why silencing the mesenchymal genes such as *CD99L2*, *EMP3*, *ITGA5*, and *VIM* alone did not lead to any significant up-regulation of E-cadherin. The effects of E-cadherin induction seen in nintedanib in combination with siRNAs against *CD99L2*, *EMP3*, *ITGA5*, *VIM* resulted from the BIBF1120 effect on the down-regulation of *SYDE1* and *ZEB1*. Targeting *ZEB1*, a transcription factor, has been demonstrated by delivering its specific targeting micro-RNA mimics via nanoliposomes [29]. However, the strategy to target *SYDE1* is still elusive, as the understanding of its role(s) in human cancers is very limited.

In conclusion, we explored two strategies to ascertain the utility of EMT reversal in carcinoma: the first, assessing EMT-related biological functions, such as the induction of colony compaction; the second, inducing re-differentiation of mesenchymal-like cells by the up-regulation of epithelial markers, such as E-cadherin. We also reported, for the first time, that a triple angiokinase inhibitor, nintedanib, exerts EMT reversal effects in both colony compaction and restoration of E-cadherin expression via the regulation of two mesenchymal genes, *SYD*E1 and *ZEB1*. The non-redundant nature of these two strategies provides an *in vitro* screening platform for the study of EMT-related functions and the utilization of selected EMT markers for drug discovery.

## MATERIALS AND METHODS

### Survival correlation of six mesenchymal signature genes in various cancer datasets

Microarray gene expression data with survival information for breast, colorectal, gastric, lung, ovarian, and pancreatic cancers were extracted from the previously processed dataset [10]. Briefly, the data were first downloaded from Gene Omnibus (GEO; http://www.ncbi.nlm.nih.gov/geo/) or ArrayExpress (https://www.ebi.ac.uk/arrayexpress/). Second, the data were RMA-normalized, and finally, combined and standardized using ComBat. In each dataset, the expression values of the six mesenchymal signature genes were converted into ranks and, subsequently, the average of the ranks was computed. The median of the average was used to define epithelial-like (< median) and mesenchymal-like (≥ median). A Kaplan-Meier analysis was performed to measure the correlation with overall and progression/disease-free survival (PDFS). PDFS includes progression-free, local recurrence-free, and distant recurrence-free survival. A log-rank test was used to assess significance of survival curve differences.

### *In vitro* culture of ovarian cancer cell line

The human OC cell line, SKOV3 (ATCC, Manassas, VA, USA), was maintained in complete high-glucose Dulbecco's modified Eagle's medium (DMEM) (Nacalai Tesque, Kyoto, Japan), supplemented with 10% (v/v) fetal bovine serum (FBS) (Biowest SAS, Nuaillé, France) and 1% penicillin and streptomycin; the cells were cultured at 37°C in a humidified atmosphere containing 5% CO_2_ and 95% air.

### Nucleic acid isolation

RNA was isolated based on the protocols described in the miRNeasy Kit (#217004, Qiagen, Valencia, CA, USA). Briefly, cells were lysed in QIAzol reagent (800 μL/10 cm^2^ culture surface area), and the lysate vigorously mixed with 160 μl of chloroform before separating the aqueous phase via centrifugation (12,000 × g, 15 min, 4°C). RNA was precipitated with 1.5× volume of absolute ethanol (of aqueous phase) and purified using the spin columns (provided in the kit). Isolated RNA was subsequently resuspended in nuclease-free water (provided in the kit). RNA concentration was determined using the NanoDrop 1000 Spectrophotometer (Thermo Fischer Scientific, Waltham, MA, USA) and RNA integrity was analyzed using the Agilent Bioanalyzer 2100 (Agilent Technologies, Santa Clara, CA, USA).

### Protein extraction and western blotting

Protein was isolated on ice using cold RIPA buffer (#R0278, Sigma-Aldrich, St Louis, MO, USA) supplemented with protease (#539134) and phosphatase (#524625) inhibitor cocktails (Calbiochem, San Diego, CA, USA). Protein concentration was quantitated using the BCA Protein Assay Kit (#23225, Thermo Fischer Scientific). SDS-PAGE electrophoresis was performed using 7.5% acrylamide gels that were transferred onto PVDF membranes (#IPFL00010; Millipore, Billerica, MA, USA). Immunoblotting was performed by blocking the membranes with 5% skim milk (Nacalai Tesque) diluted in Tris-buffered saline (TBS), followed by incubating with mouse monoclonal anti-E-cadherin (#610182, BD Biosciences, Franklin Lakes, NJ, USA; 1:2500) or mouse monoclonal anti-β-actin (#A1978, Sigma-Aldrich; 1:5000). Following washing steps, the membranes were then incubated with IRDye 800CW-conjugated (#926-32210) or IRDye 680-conjugated (#926-32220; LI-COR Biosciences, Lincoln, NE, USA) goat anti-mouse antibodies. Following final washing steps, the western blots were scanned using an Odyssey Infrared Imaging System (LI-COR Biosciences).

### MTS assays for transient knockdown of six mesenchymal signature genes

ON-TARGETplus (OTP) smartpool siRNAs for the six mesenchymal signature genes (Dharmacon Research, Inc., Lafayette, CO, USA) were custom designed. siRNAs targeting epithelial and mesenchymal genes were used to study the gene knockdown effect on Intermediate Mesenchymal SKOV3 OC cell lines, respectively. KDalert^TM^ GAPDH Assay Kit (#AM1639, Life Technologies, Carlsbad, CA, USA) was used to optimize the transfection conditions and efficiency in each cell line according to the manufacturer's protocol before the actual experiment. An OTP-none targeting pool was used as the negative control. siRNAs were transfected into each cell line in triplicate via reverse transfection concomitant with cell seeding in 96-well plates on Day 1. In brief, 50 nM of siRNA was pre-incubated with 0.3 μl of DharmaFECT 3 transfection reagent for 30 min, and then seeded onto cultures of 4000 SKOV3 cells per well, respectively. At 72-h post-transfection, the cell proliferation rate was determined using an MTS assay (#G5430, CellTiter 96^®^ Aqueous NonRad Proliferation Assay; Promega, Fitchburg, WI, USA), according to manufacturer's protocol.

### Colony compaction assays for transient knockdown of 6 mesenchymal signature genes

KDalert^TM^ GAPDH Assay Kit (#AM1639, Life Technologies) was first used to optimize the transfection conditions and efficiency in SKOV3 in a 24-well format, according to manufacturer's instructions. Reverse transfection was performed as described above with some modifications: 50 nM of siRNA with 0.4 μl DharmaFECT 3 transfection reagent was used on 5000 SKOV3 cells per well. Cells were seeded onto 33-mm diameter coverslips and incubated for 72 h before fixation with 4% paraformaldehyde (#15714S Paraformaldehyde 32% solution, Electron Microscopy Sciences, Hatfield, PA, USA). Immunofluorescence staining was performed for E-cadherin (#610182, BD Biosciences; 1:100) and DAPI (#H-1200 Vectashield Mounting Medium for fluorescence with DAPI, Vector Laboratories, Inc., Burlingame, CA, USA).

### Analysis of inter-nuclear distance

SKOV3 cells grown, fixed, and stained with DAPI on coverslips were used for the inter-nuclear distance analysis. To measure the inter-nuclear distance, random images of the cell colonies were captured at 40× magnification using an upright microscope (Zeiss Axio Imager M2; Zeiss Microimaging; Thornwood, NY, USA). The inter-nuclear distance was measured from the center of one nucleus to that of a neighboring/adjacent nucleus. The distance between the two nuclei was measured by selecting ‘Distance Measure’ option in the ZEN software (Zeiss Microimaging). A minimum of 200 inter-nuclear distances were taken to obtain the mean inter-nuclear distance for each condition.

### Annexin-V analysis for anoikis resistance assay

Cells were seeded in 6-well ultra-low attachment plates and transfected with siRNAs against the six mesenchymal genes and control siOTP genes. After 48 and 96 h of incubation, the cells were collected and trypsinized into single cell suspensions. The cells were then washed twice with PBS and counted using a hemocytometer. Equal numbers of cells were taken from each siRNA and siOTP genes and then transferred into a 15-ml Falcon tube (BD Biosciences). The cells were resuspended in 100 μl of 1× Annexin binding buffer. Five μl of Pacific Blue Annexin-V (Sigma-Aldrich) and 1 μl of 100 μg/ml Propidium Iodide (Sigma-Aldrich) working solution were added and incubated for 15 min at room temperature. Data were acquired using a BD LSRII flow cytometer (BD Biosciences).

### Cloning of promoter constructs of CDH1 and CDH3

The putative 1.2-kb promoter regions of CDH1 and CDH3 around the transcriptional start site (TSS)—ranging from −1.0-kb to +0.2-kb of the TSS (Supplementary Table S1)—were first cloned from the genomic DNA of OVCA420 and HEY cells using PCR. The correct fragments were confirmed and purified using agarose gel electrophoresis. The gel-purified fragments were then digested using restriction enzymes (RE) and column-purified before cloning into a pGL3basic luciferase-expressing vector (#E1751, Promega) in *E. coli* competent cells (#C4040-03 One Shot^®^ TOP10 Chemically Competent *E. coli*, Life Technologies). Positive clones were selected and further confirmed by sequencing. The cloning sequences, cloning primers, and RE sites are summarized in Supplementary Table S1.

### *In vitro* EMT reversal assays

Cells of interest were grown in complete media in either 6-well plates (#140675, Nunc, Denmark) or 100-mm dishes (#150350, Nunc) to allow growth until 60% confluence prior to drug treatment. DMSO (#D8418, Sigma-Aldrich; 0.05%), as a control, or nintedanib (BIBF1120) at various concentrations were added for 24 h or 3 days (without replenishment) and the cells were then subjected to downstream assays. Cells were examined under a light microscope with phase contrast rings (Olympus Optical Co. Ltd, Tokyo, Japan) to document morphological changes. RNA and protein were harvested and subjected to QPCR analysis and western blotting, respectively, as described above. For promoter assays, cells were seeded into 96-well plates (#3904, Corning Inc., Corning, NY, USA) at a density of 0.5–1.0×10^4^ cells per well. After 24 h, the cells were transfected with promoters of EMT signature genes or vector control plasmid using XtremeGENE HP (#6366236001, Roche Applied Science; Indianapolis, IN, USA) with a 2:1 HP:DNA ratio. The cells were treated with each drug on Day 3 at a final concentration of 5 μM per well. The dual luciferase assay (#E1960, Promega) was conducted on Day 4, according to manufacturer's protocol.

### Gene expression microarray analysis for EMT scores and gene ontology (GO)

Microarray gene expression data of SKOV3 cells (GFP- and drug-treated) were RMA-normalized. A log-2 fold-change relative to SKOV3-GFP was subsequently computed for each gene and for each drug treatment. Differentially expressed genes with log-2 fold-change relative to SKOV3-GFP that were consistently greater than +1.5 or lesser than −1.5 among replicates were deemed significant. Gene ontology analysis was conducted using the significant differentially expressed genes and DAVID v6.7 (http://david.abcc.ncifcrf.gov/summary.jsp). False-discovery rate less than 25% was deemed significant.

### Cell cycle analysis

SKOV3-GFP cells were synchronised by serum starvation in 10-cm dishes. After synchronization, the serum-free medium was removed and the cells were washed with 1×PBS and replenished with fresh medium containing DMEM with 10% FBS together with the TKIs or vehicle control. After 48 h, the cells were harvested, washed with ice-cold PBS and fixed with ice-cold 70% ethanol overnight at 4°C. The cells were then centrifuged at 600 ×*g* for 10 min at 4°C, and the ethanol discarded. The cells were washed twice with ice-cold PBS. The cells were then counted, and equal numbers of cells from each condition were transferred into 5-ml round tubes. The cells were treated with 10 μg/ml RNase A (Roche Applied Science) for 10 min at room temperature and then stained with 200 ng/ml of Propidium Iodide (Sigma-Aldrich) in the dark for 15 min. Cell cycle analysis was performed using BD LSR II (BD Biosciences) and analysed using the FlowJo flow cytometry analysis software (Tree Star, Inc., Ashland, OR, USA).

### GI50 assay of mitotic inhibitors

SKOV3-GFP cells were tested for their sensitivity to paclitaxel, docetaxel and vincristine with BIBF1120. Paclitaxel (#T7402) and vincristine (#V8879) were purchased from Sigma-Aldrich and docetaxel was provided by Dr Wang Ling Zhi at Cancer Science Institute of Singapore. The cells were seeded in 96-well plates at an optimal density pre-determined to ensure that 80% confluence was attained by the end of the assay. Following an overnight incubation, the cells were treated with either DMSO (vehicle control) or 5 μM BIBF1120 for 24 h, then nine concentrations of each drug (two-fold dilution series over a 128-fold concentration range) and co-treated with either DMSO or 5 μM BIBF1120 for 48 h on the following day. The percentage of the cell population responding to the drug relative to the negative controls was measured using a CellTiter 96 AQueous Non-Radioactive Cell Proliferation Assay, following the manufacturer's recommendations (#G5430, Promega). Dose-response curves were plotted using GraphPad Prism^®^ version 5.04 (GraphPad Software, Inc., La Jolla, CA, USA), to derive a growth inhibitory concentration of 50% (GI50; drug concentration for 50% growth inhibitory effects on cells) for each drug treatment in at least three independent experiments. A Mann-Whitney U-test of GraphPad Prism was used to statistically evaluate the averaged GI50s between SKOV3-GFP DMSO-treated and SKOV3-GFP BIBF1120-treated cells.

### SKOV3 xenograft experiments for EMT reversal

All animal work adhered to the Agency for Science, Technology and Research (A*STAR; Singapore), Institutional Animal Care and Use Committee (IACUC) guidelines on the use and handling of animals. SKOV3-Luc-D3 cells (Xenogen Co., Alameda, CA, USA) at a density of 3.5×10^6^ in 100 μl of PBS were injected into the intraperitoneal cavity of 4-week-old female BALB/c nude mice. At 6 weeks post-implantation, the mice were randomly divided into control and treatment groups (*n* = 5 animals per group). For the treatment group, mice were administered via oral gavage with 50 mg/kg AZD0530, 50 mg/kg BIBF1120, or 25 mg/kg AZD0530 plus 25 mg/kg BIBF1120 (Selleck Chemicals, Houston, TX) for 5 days a week for 2 weeks. The drug was re-suspended in 0.5% hydroxypropyl methycellulose (Sigma-Aldrich) and 0.1% polysorbate buffer (Sigma-Aldrich). The control group received the vehicle buffer alone. The growth of tumor xenografts was monitored by bioluminescence using the IVIS system 2000 series (Xenogen Co.). The xenografts were harvested at 8 weeks post-implantation for gene expression microarray analysis (Affymetrix GeneChip Human Gene 1.0ST Array, Santa Clara, CA, USA) to ascertain the EMT scores, and then subjected to paraffin embedding followed by immunohistochemical staining for E-cadherin (#3195S, Cell Signaling Technology, Beverly, MA).

## SUPPLEMENTARY MATERIAL FIGURES AND TABLE


