# Supplementary material for: Functional relevance of a six mesenchymal gene signature in epithelial-mesenchymal transition (EMT) reversal by the triple angiokinase inhibitor, nintedanib (BIBF1120)
Source: Oncotarget. 2015 May 27;6(26):22098–113. doi: 10.18632/oncotarget.4300 (PMC4673149; doi:10.18632/oncotarget.4300)
Supplement: Supplementary file 1 [file oncotarget-06-22098-s001.pdf]

Functional relevance of a six mesenchymal gene signature in epithelial-mesenchymal transition (EMT) reversal by the triple angiokinase inhibitor, nintedanib (BIBF1120)

Supplementary Material

Materials and Methods

Ovarian carcinoma cell line library

An ovarian carcinoma cell line library, termed SGOCL(43) [1], comprising 43 different ovarian carcinoma cell lines of serous, endometrioid, and undifferentiated histology, was acquired via various sources and maintained in house. Detailed cell line names and growth conditions used in this study have been described elsewhere [1].

Quantification of 33 EMT signature genes in ovarian carcinoma cell lines

SGOCL(43) cell lines were grown in 100-mm tissue culture plates (Corning Inc., Corning, NY, USA) until 90% confluence before harvesting. Each cell line was grown in duplicate. RNA was extracted as described above using Qiazol (Qiagen, Valencia, CA, USA) and prepared for real-time PCR using the miRNeasy columns (#217004, Qiagen). RNA (500 ng) was reverse transcribed into cDNA using the RT^2 First Stand Synthesis Kit (Qiagen) and then subjected to real-time quantitative PCR analysis for 33 EMT signature genes (Qiagen RT2 qPCR Primer Assay). The reactions were carried out according to the manufacturer's protocol on a 7900HT Fast Real-Time PCR System (Applied Biosystems, Foster City, CA, USA) using the RT^2 qPCR SYBR/ROX Master Mix and equal volumes of mixtures. Five housekeeping genes (*ACTB*, *B2M*, *GAPDH*, *HPRT1* and *RPL13A*) were used for standardization and five assay quality controls (one human genomic DNA contamination, two reverse transcription, and two positive PCR controls) were employed. Data in the form of threshold cycle numbers (C_t) were uploaded to the online data analysis portal (http://pcrdataanalysis.sabiosciences.com/pcr/arrayanalysis.php) to calculate the delta-C_t (ΔC_t). C_t was determined through the SDS (version 2.3) software (Applied Biosystems) by setting the baseline between cycle 2 of the run (total run: 40 cycles) and two cycles before the start of the first log-phase amplification. The threshold was set by positioning the limit to the lower one-third of the earliest amplification. ΔC_t was calculated by the respective formula below:

$\Delta C_t = C_t\ (GOI) - C_t\ (HKG)$

where C_t (GOI) is the C_t value of the respective gene of interest (GOI) and C_t (HKG) is the average C_t values of the five housekeeping genes (HKG) used in the assay.

Immunofluorescence staining of EMT markers

Cells were grown on 15-mm glass coverslips (Paul Marienfeld GmbH & Co. KG, Lauda-Königshofen, Germany) until 70-80% confluent. Cells were then fixed in cold acetone at -20°C for 10 min, rehydrated with 1× PBS (thrice, 5 min each) and blocked with 3% (w/v) BSA (Fraction V; Sigma-Aldrich, St Louis, MO, USA) in 1× PBS for 1 h at room temperature. After washing with PBS, incubations with primary antibodies against E-cadherin (#610182, BD Biosciences; San Jose, CA, USA; diluted 1:100), DDR1 (#5583, Cell Signaling Technology Inc., Beverly, MA, USA; diluted 1:1000), and GRHL2 (#HPA004820, Sigma-Aldrich; diluted 1:500) were performed at 37°C for 1 h. After washing with PBS, incubations with secondary antibodies conjugated with Alexa-488 (#A11029, #A11034, Invitrogen, Carlsbad, CA, USA) were performed at room temperature for 1 h in the dark. Slides were washed again with PBS, and coverslips were subsequently mounted with anti-fading mounting media (Vector Laboratories Inc., Burlingame, CA, USA). All images were viewed on an Olympus IX71 fluorescent microscope and images were taken under 10× magnification using the Olympus DP71 camera (Olympus Optical Co. Ltd, Tokyo, Japan).

Combination effect of AZD0530 and BIBF1120

The effect of AZD0530-BIBF1120 combination on E-cad promoter activity was assessed based on Chou-Talalay combination index (CI) [2],

$$\mathrm{CI} = \frac{D_{\mathrm{AZD0530},x}}{D_{x,\mathrm{AZD0530}}} + \frac{D_{\mathrm{BIBF1120},x}}{D_{x,\mathrm{BIBF1120}}}$$

where $D_{x,\mathrm{A}}$ denotes concentration of Drug A alone that produce effect or fraction affected (Fa) of x, whereas $D_{\mathrm{A},x}$ is the concentration of Drug A in combination (with Drug B) that produce the same effect x. Based on CI value, synergistic (CI<1), additive (CI=1), or antagonistic (CI>1) drug-drug interaction can be determined.

To compute CI, SKOV3 cells were treated with DMSO or 5 concentrations (0.0625, 0.125, 0.25, 0.5, 1.0μM) of AZD0530 each in combinations with 5 concentrations (0.1875, 0.375, 0.75, 1.5, and 3μM) of BIBF1120. Each drug was also used alone at these concentrations. These combinations yielded a 6 × 6 table. Effect or Fa for each drug-drug combinations is measured by E-cad promoter activity fold change relative to DMSO averaging over quadruplicate experiments, and normalized to maximum achievable fold change in these combinations. The table was subsequently used for computation and analysis of CI using CompuSyn version 1.0 (ComboSyn Inc.; Paramus, NJ) [3] and Graphpad Prism ® version 5.04 (GraphPad Software; La Jolla, CA).

Results

Good correlations exist between the six-mesenchymal gene signature and the transitioned phenotypes

We validated the expression levels of the 33 genes from the EMT signature (Huang et al., 2013) on a panel of ovarian cancer (OC) cell lines, SGOCL(43), using quantitative PCR (QPCR). As shown in the heatmap (Supplementary Figure S 1A), the transcript expressions of these 33 EMT signature genes correlated well with the EMT phenotypes identified by the EMT spectrum, with Epithelial-classified cell lines showing the highest expression levels for epithelial genes. We checked the protein expression levels of two epithelial genes, *DDR1* (Supplementary Figure S1 B) and *GRHL2* (Supplementary Figure S1 C), using western blotting and immunofluorescence (IF) in selected epithelial-like and mesenchymal-like OC cell lines. Both proteins were preferentially enriched in epithelial-like cell lines and were absent in mesenchymal-like cell lines. Among the six designated mesenchymal genes (*EMP3*, *ITGA5*, *ZEB1*, *VIM*, *CD99L2*, *SYDE1*) [1], *CD99L2* and *SYDE1* were clustered in between the epithelial genes, which was reflected in their up-regulation in the partially transitioned or 'EMTed' Intermediate E cells (*CD99L2* and *SYDE1* would still be described herein as "mesenchymal" genes).

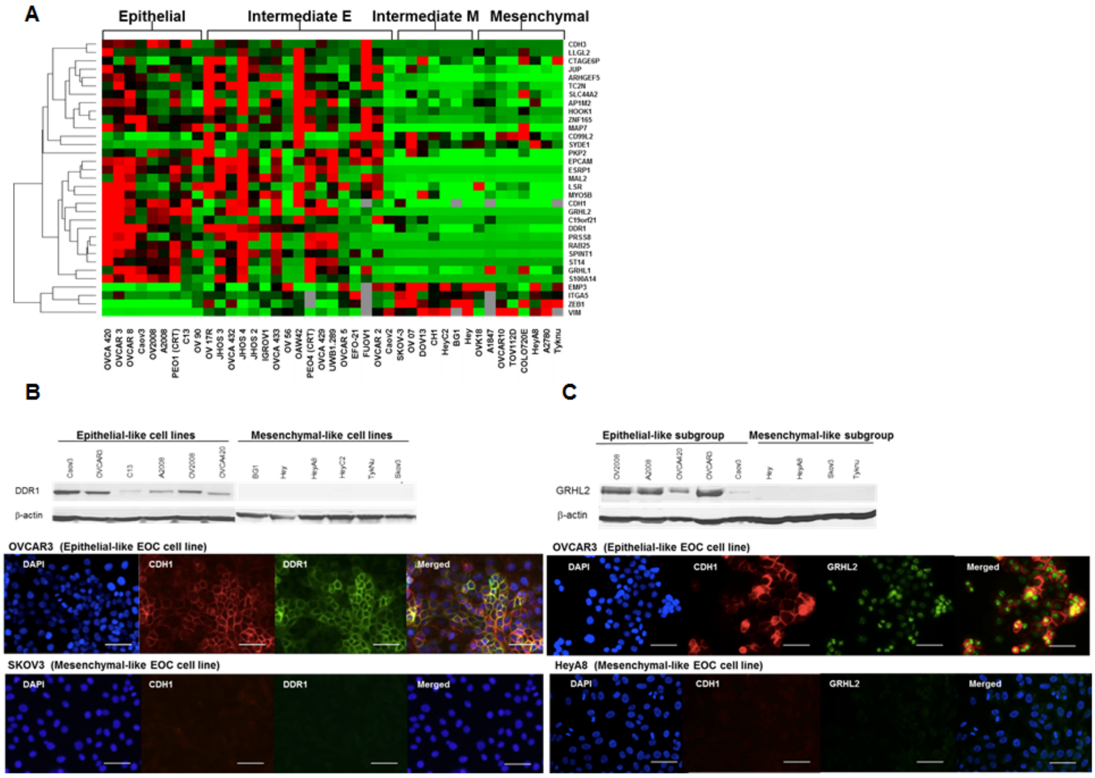

Supplementary Figure 1: Validation of a 33-gene EMT signature in SGOCL(43). (A) Heatmap of the clustering of QPCR expression (2^- Avg ΔCt) of 33 EMT signature genes. (B) Western Blotting of discoidin-domain receptor 1 (DDR1) and β-actin in epithelial-like (Caov3, OVCAR3, C13, A2008, OV2008, OVCA420) and mesenchymal-like (BG1, Hey, HeyA8, HeyC2, TykNu, SKOV3) ovarian cancer cell lines. Immunofluorescence (IF) staining of E-cadherin (E-cad; red) and DDR1 (green) in OVCAR3 and SKOV3 cells. Nuclei were counterstained with DAPI (blue). (C) Western Blotting of grainy-head-like 2 (GRHL2) and β-actin in epithelial-like (OV2008, A2008, OVCA420, OVCAR3, Caov3) and mesenchymal-like (Hey, HeyA8, SKOV3, TykNu) cells. IF staining of CDH1 (red) and GRHL2 (green) in OVCAR3 and HeyA8 cells. Bar: 50 µm.

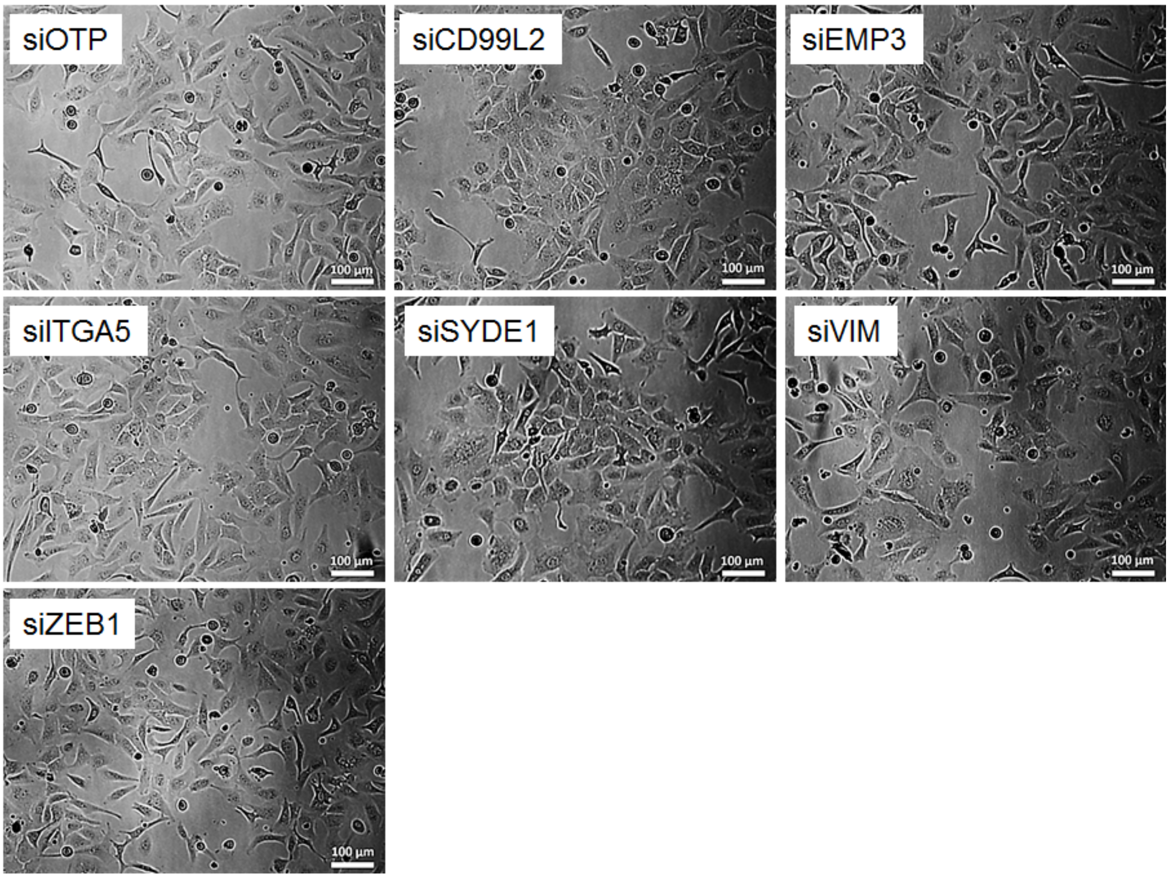

Supplementary Figure 2: Phase contrast images of SKOV3 cells following transient silencing of the six mesenchymal genes (siCD99L2, siEMP3, siITGA5, siSYDE1, siVIM, siZEB1) and si-OTP control. Bar: 100 µm.

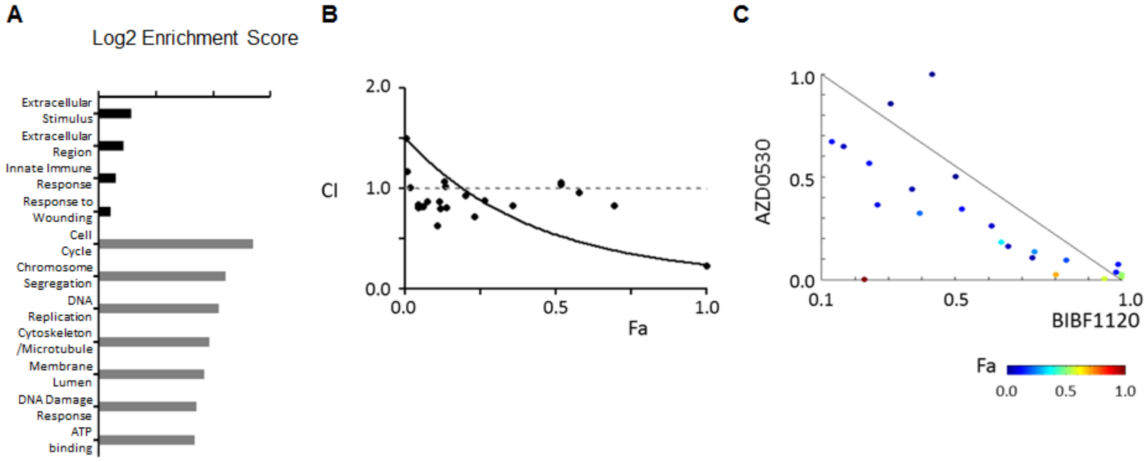

Supplementary Figure 3: Effects of combination of AZD0530 and BIBF1120. (A) Bar chart of the log2 enrichment score of the gene ontology (GO) analysis of TKI-treated SKOV3-GFP cells. (B) Fraction-affected (Fa; *x*-axis)-Combination index (CI; *y*-axis) plot. Fa is the E-cad promoter activity fold change relative to DMSO averaged over quadruplicate, and normalized to highest achievable fold change in the experiments. Trendline was fitted using one-phase decay exponential curve with constraint. CI = 1, additivity; CI>1, antagonism; CI<1, synergy. (C) Normalized Isobologram for AZD0530 (*y*-axis) and BIBF1120 (*x*-axis) combination. The black line indicates additivity, and the dots show the combination ratios producing different Fa (blue: low Fa%, maroon: high Fa%). Data below and above the line of additivity indicate synergy and antagonism respectively.

Supplementary Table 1. Summary of cloning sites, sequences, and primers for CDH1, CDH3, and E-cadherin (E-cad) promoters.

No.	Symbol	Cloning sites	Promoter Region	Forward primer	Reverse primer
1	CDH1	KpnI, HindIII	-1085 to +99	GGGGTACCCTAACCCATGAAGCTCTACAG	CCTTAAGCTTGCTCACAGGTGCTTTGCAGTTCC
2	CDH3	KpnI, BglII	-1017 to +214	GGGGTACCTCCAGTCTCCAGTCCGTTC	GAAGATCTCCTCCTCAGTACGGGAAG
3	E-cad	SacI, HindIII	-108 to +125	-	-

References

1. Huang RY, Wong MK, Tan TZ, Kuay KT, Ng AH, Chung VY, Chu YS, Matsumura N, Lai HC, Lee YF, Sim WJ, Chai C, Pietschmann E, Mori S, Low JJ, Choolani M, et al. An EMT spectrum defines an anoikis-resistant and spheroidogenic intermediate mesenchymal state that is sensitive to e-cadherin restoration by a src-kinase inhibitor, saracatinib (AZD0530). Cell Death Dis. 2013; 4: e915.

2. Chou TC (2010) Drug combination studies and their synergy quantification using the Chou-Talalay method. Cancer Res 70: 440–6

3. Chou, T.-C. and Martin, N (2007) CompuSyn software for drug combinations and for general dose effect analysis, and user’s guide. ComboSyn, Inc. Paramus, NJ.
